# In Vitro & In Vivo Anti-Hyperglycemic Potential of Saponins Cake and Argan Oil from *Argania spinosa*

**DOI:** 10.3390/foods10051078

**Published:** 2021-05-13

**Authors:** Rabie Kamal, Mourad Kharbach, Yvan Vander Heyden, Huiwen Yu, Abdelaziz Bouklouze, Yahia Cherrah, Katim Alaoui

**Affiliations:** 1 Pharmacodynamic Research Team ERP, Laboratory of Pharmacology and Toxicology, Faculty of Medicine and Pharmacy, Mohammed V University, Rabat BP 6203, Rabat Instituts, Rabat 10100, Morocco; rabie1kamal@gmail.com (R.K.); y.cherrah@um5s.net.ma (Y.C.); k.alaoui@um5s.net.ma (K.A.); 2 Clinical Research Management (MBA), IU–International University of Applied Sciences, Kaiserplatz 1, 83435 Bad Reichenhall, Germany; 3 Department of Analytical Chemistry Applied Chemometrics and Molecular Modelling, CePhaR, Vrije Universiteit Brussel (VUB), Laarbeeklaan 103, B-1090 Brussels, Belgium; Mourad.Kharbach@oulu.fi (M.K.); Yvan.Vander.Heyden@vub.be (Y.V.H.); 4 Research Unit of Mathematical Sciences, University of Oulu, FI-90014 Oulu, Finland; 5 Chemometrics and Analytical Technology, Faculty of Science, University of Copenhagen, Rolighedsvej 26, 1958 Frederiksberg, Denmark; 6 Bio-Pharmaceutical and Toxicological Analysis Research Team, Laboratory of Pharmacology and Toxicology, Faculty of Medicine and Pharmacy, University Mohammed V, Rabat BP 6203, Rabat Instituts, Rabat 10100, Morocco; a.bouklouze@um5s.net.ma

**Keywords:** argan saponins cake, argan oil, in-vivo hyperglycemic activity, in-vitro hyperglycemic activity, α-glucosidase inhibition, α-amylase inhibition

## Abstract

The Argan tree (*Argania spinosa*. L) is an evergreen tree endemic of southwestern Morocco. For centuries, various formulations have been used to treat several illnesses including diabetes. However, scientific results supporting these actions are needed. Hence, Argan fruit products (i.e., cake byproducts (saponins extract) and hand pressed Argan oil) were tested for their in-vitro anti-hyperglycemic activity, using α-glucosidase and α-amylase assays. The in-vivo anti-hyperglycemic activity was evaluated in a model of alloxan-induced diabetic mice. The diabetic animals were orally administered 100 mg/kg body weight of aqueous saponins cake extract and 3 mL/kg of Argan oil, respectively, to evaluate the anti-hyperglycemic effect. The blood glucose concentration and body weight of the experimental animals were monitored for 30 days. The chemical properties and composition of the Argan oil were assessed including acidity, peroxides, K232, K270, fatty acids, sterols, tocopherols, total polyphenols, and phenolic compounds. The saponins cake extract produced a significant reduction in blood glucose concentration in diabetic mice, which was better than the Argan oil. This decrease was equivalent to that detected in mice treated with metformin after 2–4 weeks. Moreover, the saponins cake extract showed a strong inhibitory action on α-amylase and α-glucosidase, which is also higher than that of Argan oil.

## 1. Introduction

Type 2 diabetes mellitus (T2DM) and hypertension are the most shared comorbidities in coronavirus-infected patients [1]. In the current COVID-19 pandemic context and according to some papers [1,2], including those from the Centers for Disease Control and Prevention (CDC), patients with type 2 diabetes mellitus and the metabolic syndrome could suffer with an up to ten-times higher risk of dying when they contract COVID-19 [3].

T2DM is a universal disease affecting the populations of developed and developing nations. Moreover, T2DM is the most common endocrine disease with indirect relation to several other disorders. It is expected that more than 300 million persons worldwide will suffer from T2DM in 2025 [4]. A genetic susceptibility to the disease exists that is promoted by environmental reasons, for example an unhealthful eating behavior, with obesity being one of the greatest important risk reasons. T2DM is caused by an irregularity of the carbohydrate metabolism, which is directly connected to down insulin levels in blood [5,6].

Recently, natural food components and natural anti-hyperglycemic mixtures, which have advantages on human health, have received additional interest from the scientific society [7]. For example, the ethanolic extract of Kigelia Africana indicated the existence of bioactive compounds like phenolic acids and iridoids with beneficial activity in insulin resistance and diabetes, and other bioactive compounds in Kigelia Africana presented protective effects for patients with testis cancer against the damages caused by chemotherapy [8].

Some experiments were performed on the anti-hyperglycemic impacts of Mediterranean foods and plants [9,10,11]. The anti-hyperglycemic effect was analyzed in directive to show effective, safe, and natural components for pharmaceutical applications and food diets [7]. The saponin compounds and the oil from many plants exhibit an anti-hyperglycemic action that can be used against diabetes and its long-term complications [12]. No study has been reported yet on the in-vitro and in-vivo anti-hyperglycemic activities of *Argania spinosa* (saponins and oil).

A study has proposed that the dietary intake of edible oils, for example, olive oil, containing phenolic compounds, can decrease chronic inflammatory disease progress [7].

Another work, about the structure of some extra virgin oils formed by cold pressing (macadamia, pequi, avocado, palm, coconut, and olive), suggested important levels of natural antioxidants marked some of the considered oils which could be categorized by a better product safety, and by good health properties [13]. The beneficial effects of extra virgin olive oil (EVOO) on human health are related to its high nutritional value, and its distinctive composition in fatty acids and minor elements, like phytosterols and squalene, polyphenols, aldehydes, pigments, minerals, and vitamins [14].

Different biological properties generally recognized by traditional medicine have obtained several scientific endorsements. This is particularly the situation for the traditional utilization of almond oil to reduce hypertension and blood cholesterol level [15,16,17], to cure skin diseases like chicken pox pustules and juvenile acne, or for the more beneficial activities of saponin derivatives especially their anti-microbial, and lipolytic properties [18]. The antioxidant properties of saponins from A. spinosa and their capability to potentate the antioxidant activity of vitamin E were also demonstrated [19].

**Figure 1:**
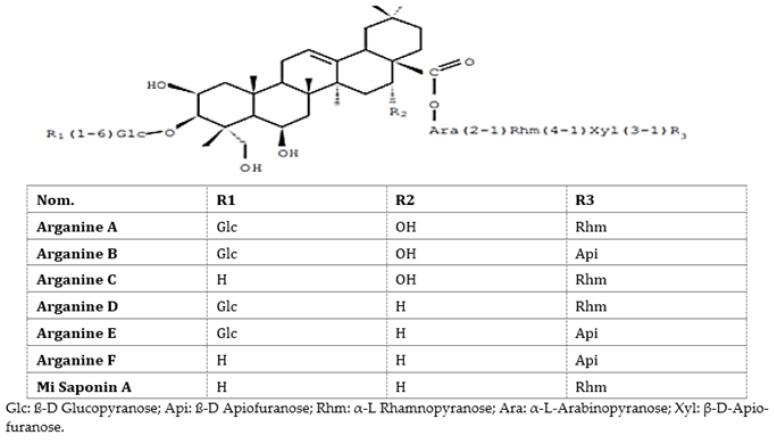
Chemical structure of *Argania spinosa* saponins [21]. Copyright © 1992 Published by Elsevier Ltd.

Earlier, our team evaluated the toxicity and pharmacological activities of saponins of A. spinosa cakes in mice and rats [20]. Argan cake saponins (Figure 1) were found not toxic orally (lethal dose; DL 1300 mg/kg per os) and showed at 50 mg/kg per os a peripheric analgesic action equivalent to acetyl salicylic acid (200 mg/kg per os). A total safety was acquired with 500 mg/kg per os. Anti-inflammatory experiments were performed in vivo utilizing oedema due to carrageenan or experimental trauma in mice. There was a reduction in the foot inflammation at 10 mg/kg per os. At doses of 50 to 100 mg/kg per os, the anti-inflammatory action was comparable to that of indomethacin at 10 to 20 mg/kg per os. The chemical structures of *Argania spinosa* saponins are presented in Figure 1.

Many chemical analyses discovered that Argan oil is principally well stable in relation to its fatty acid composition [22,23,24,25].

We consequently studied the anti-hyperglycemic effect of Argan seeds by researching the actions of saponin extracts using α-glucosidase and α-amylase assays as well as an in-vivo model of alloxan-induced diabetic mice. In particular, we evaluated the ability of Argan extracts to rise the inhibitory properties on digestive enzymes (α-amylase and α-glucosidase). The saponin extracts had an activity with an antidiabetic potential.

The specific chemical profile of the Argan fruit extracts, namely cake and Argan oil, could be the reason of a possible anti-hyperglycemic action. The chemical composition and bioactive molecules were discussed. This experiment presents and discusses the first study about the in-vitro and in-vivo antidiabetic potential of *Argania spinosa* saponins cake extracts and oil.

## 2. Materials and Methods

### 2.1. Sample Preparation and Extraction

Recently, we reported that traditional Argan oil and cake saponins had several pharmacological activities [7,20,26,27].

The sample collection was from the cooperatives of Amanar (Morocco), which extracts Argan oil from kernels harvested in the Argan grove in Taroudant region (southwestern Morocco). Argan fruits were collected in the summer of 2016 and Argan kernels were ready in February 2017. The samples were prepared by extraction of roasted Argan kernels at 110 °C for 25 min. From the same kernels, edible traditional Argan oil and saponin cakes of *Argania spinosa* were obtained according to the technique described by Alaoui et al. [26]. The kernels (1 kg) of the Argan fruit were reduced to a fine powder and successively extracted with hexane and ethanol/water 80-20 (*v*-*v*) by Soxhlet extraction. The hydroalcoholic extract was concentrated under decreased pressure. This residue was suspended in water treated two or three times with ether, and then extracted twice with n-butanol. The aqueous phase was exhausted. The butanolic phase was decanted and evaporated under lowered pressure to dryness. The dry residue was retaliated with a minimum of methanol and distilled water and then lyophilized in a Telstar Cryodos Lyophilizator. This gave a yellowish-white, amorphous powder, soluble in water, containing the mixture of saponins. Then, saponin aqueous extract with a concentration of 100 mg/kg was administered to adult Swiss albino mice.

#### 2.1.1. Physicochemical Quality Evaluation

In this study the hand pressed Argan oil was examined controlling chemical bioactive parameters.

The oil free acidity (% oleic acid), peroxide value (PV, milli-equivalents of active oxygen per kilogram of oil (meq O_2_/kg)), and absorbance coefficients K270 (270 nm) and K232 (232 nm), were measured according to the standard methods described in EEC/2568/91 of the European Commission Regulations [28].

#### 2.1.2. Fatty Acid and Sterol Determination

The fatty acids (FA) were determined via a methyl-trans-esterification, fatty acid methyl esters (FAMEs) of the Argan oil, following the procedure described by the Official Method for FA analysis [28]. The analytical conditions are also described in Kharbach et al. [23]. The FAMEs were separated by gas chromatography (6890/Agilent Technologies, Wilmington, DE, USA) and measured with a flame ionization detector (FID). The chromatographic separation was carried out on a capillary column (100 m × 0.25 mm ID × 0.20 µm film) (HP-88, Agilent Technologies Spain, Madrid) with a 1 mL/min flow rate of Helium carrier gas. The following temperatures were maintained: column oven 210 °C, injector 230 °C, and detector 250 °C, respectively. Eight individual fatty acids were identified and quantified using fatty acid standards (Sigma-Aldrich, Lyon, France). Each FA content was expressed as a percentage (g/100 g oil).

The sterol composition was measured according to the official method for individual and total sterols contents described by the NP ISO 12228:2002 [29]. A gas chromatograph (6890-Agilent Model Technologies, Wilmington, DE, USA) equipped with a flame ionization detector (FID) was used for sterol separation. A DB-5 capillary column (30 m × 0.25 mm ID × 0.25 µm film) (Agilent Technologies, Madrid, Spain) was selected to perform this separation, applying the conditions described by Kharbach et al. [23]. Briefly, the column temperature was 280 °C, injector temperature 290 °C, and the detector was set at 290 °C. Helium was used as the carrier gas, with a flow rate of 2 mL/min. Each sterol amount was expressed as percentage (%), while the total sterols were expressed as mg/100 g of Argan oil. Sterol standards (Sigma-Aldrich, Lyon, France) were used to identify and quantify 6 sterols in Argan oil. All measurements were repeated in triplicate.

#### 2.1.3. Tocopherol Determination

Four individual tocopherols (α, β, δ and γ- tocopherol) were quantified using Method Ce8-89 described by AOCS [30]. This procedure was earlier also described in Kharbach et al. [24]. Briefly, 10 mg of Argan oil was dissolved in n-hexane (100 mL), then injected in a liquid chromatograph (1100 series, Agilent Technologies, Waldbronn, Germany). The system was coupled to a fluorescence detector (G1321A model) operated at excitation and emission wavelengths of (λexc = 290 nm) and (λemis = 330 nm), respectively. A reversed-phase C18 column (25 cm × 4.6 mm × 5.0 µm) from ChromSpher (Varian, Middelburg, The Netherlands) was used. An acetonitrile/methanol (50:50, *v*/*v*) mixture was used for elution (mobile phase) in isocratic mode with a flow rate of 1 mL/min. A sample volume of 20 μL was injected.

The tocopherols quantification was done using the external tocopherol standard (i.e., α-, β-, γ-, and δ-tocopherol; Sigma-Aldrich, Lyon, France). The total tocopherol (or vitamin E) content was calculated as the sum of those four individual tocopherols.

#### 2.1.4. Total Polyphenols

The total polyphenol amount was quantified by the Folin–Ciocalteu colorimetric technique [31]. The polyphenols of Argan oil were extracted using a liquid–liquid extraction. An amount of 10 g oil was diluted in n-hexane (50 mL) pure solvent and extracted twice with a mixture of methanol–water (50:50, *w*/*w*). The recuperated polyphenolic extract was also washed twice by n-hexane, and lyophilized during 12 h [32]. Later, 10 mg of the extract (polyphenols) was dissolved in 10,0 mL of methanol, and then 100 μL was used with 500 μL of a fresh solution of Folin–Ciocalteu, 1000 μL milli-Q water, and 1500 μL sodium carbonate (Na_2_CO_3_, 20% *m*/*v*) [7]. This sample was incubated in the dark for 2 h. The absorbance was measured at 765 nm (Ultraviolet; UV-2450, UV–Visible spectrophotometer, Shimadzu Technologies, Tokyo, Japan). The quantification was done using the calibration curve of the gallic-acid standard, and the total polyphenols were expressed in mg GAE·kg^−1^ (mg gallic-acid equivalents per kilogram oil). This standard was purchased from Sigma-Aldrich, St Quentin Fallavier, France.

#### 2.1.5. Phenolic Composition Measurement

The phenolic profiling, identification, and quantification was achieved following the method described in Kamal et al. [7]. Briefly, a liquid chromatography instrument (Agilent System Technologies, 1100, Wilmington, DE, USA) was hyphenated to a Diode-Array Detector (DAD/G1315-B). The instrument was equipped with an autosampler (G1330-B/Agilent), and a binary pump (G1312-A/Agilent). The HPLC-DAD was interfaced to a time-of-flight mass-spectroscopic detector (TOF/MS; Agilent Technologies) by an electrospray ionizer source (ESI-; Micro-mass Quattro Micro). The mass spectra were functioned in negative mode applying the operative conditions as follows: extractor, 2 V; capillary and cone voltages were 3.0 kV and 20 V; cone gas flow 30 L·h^−1^; source temperature 100 °C; desolvation gas flow 350 L·h^−1^; and desolvation temperature 350 °C. The phenolic elution was achieved on a C18 column (Zorbax, Eclipse, XDB-C18, 2.1 mm × 100 mm, 1.7 µm particle size). The chromatographic separation was done using binary mobile phase solvents, acetonitrile with 0.1% formic acid (A) (*v*/*v*) and pure water with 0.1% formic acid (B) with a flow rate of 0.5 mL/min, and using the optimum gradient (*v*/*v*), 10% A, 0 min; 70% A, 0–18 min; 100% A, 18–20 min; 100% A, 20–23 min; 10% A, 23–25 min; and 10% A, 25–30 min. At a column temperature of 35 °C and 10 µL was injected. The phenolics quantification and identification were done using the chromatographic information (retention times, mass spectra fragmentation, and standards calibration curves). The pure standards were obtained from Sigma-Aldrich (St Quentin Fallavier, France).

The phenolic extract (Argan oil) was collected by the liquid–liquid extraction described earlier, while 10 mg of extract was dissolved in 10 mL of methanol and n-hexane was applied for successive washing (three times). Later, 1 mL of the extract was passed through a syringe filter (PVDF, 0.2 µm) and was injected in the chromatographic system.

### 2.2. In-Vitro Antidiabetic Activity

#### Amylase and Glucosidase Inhibition and Kinetics

The α-amylase inhibitory capacities were studied by reacting varying concentrations of the extracts with α-amylase and starch solution, corresponding to the technique described in [33,34]. Furthermore, 250 μL extract and 250 μL 0.02 M sodium phosphate buffer (pH = 6.9) containing α-amylase (240 U/mL) were kept during 20 min at 37 °C. Later, 250 μL of 1% starch solution in 0.02 M sodium phosphate tampon (pH = 6.9) was added to the combination, and kept during 15 min at 37 °C. Subsequently, 1 mL dinitrosalicylic acid (DNS) was added and the reaction mixture was kept in a boiling water bath for 10 min. Next, a dilution was created by making 2 mL distilled water, and the absorbance at 540 nm was measured using ultraviolet-visible (UV-Vis) spectrophotometry. The positive control was acarbose [34,35].

Exploiting the method explained by Kee et al. [36], the α-glucosidase inhibitory activity of the extracts was revealed utilizing the pNPG substrate. Next, α-glucosidase (0.1 U/mL) and p-nitrophenyl-α-D-glucopyranoside (p-NPG, 1 mM) substrate were dispersed in phosphate buffer 0.1 M, pH 6.7. The mixture of 150 μL model and 100 μL 0.1 M sodium phosphate buffer (pH = 6.7) including α-glucosidase (0.1 U/mL) was kept at 37 °C for 10 min exactly. Then, 200 μL 1 mM pNPG was combined and the enzymatic reaction was kept at 37 °C for 30 min. Later, 1 mL 0.1 M Na_2_CO_3_ was included, and the absorbance was evaluated at 405 nm. The α-glucosidase inhibitory effect was stated as a percentage inhibition and the IC50 values were defined. Acarbose was utilized as the positive control [34].

With improving concentrations of the substrate (starch and p-nitrophenyl-α-D-glucopyranoside p-NPG) in the existence of various concentrations of extracts, the type of inhibition of α-glucosidase and α-amylase was examined. Later, the kind of inhibition was decided by the Lineweaver–Burk plot analysis of the information, analyzed from the findings according to Michaelis–Menten kinetics.

The percentage inhibition is determined by this equation for the two assays:

The %Inhibition = [(Ac − Acb) − (As − Asb)/(Ac − Acb)] × 100(1)

As (absorbances sample), Ac (absorbances control), Asb (absorbances sample blank), and Acb (absorbances control blank).

### 2.3. In-Vivo Antidiabetic Activity

#### 2.3.1. Animals

The adult Swiss albino mice (25–30 g) were farmed in the mice facility of the Faculty of Medicine and Pharmacy (FMPR) of Rabat, Morocco. Mice were maintained in cages (Biobase Laboratory mouse cage) under normal laboratory environments, in a 12 h light/12 h dark cycle at a temperature of 22 to 24 °C. The structure of the mice diet is described in Table 1. The research was directed in agreement with the ethics of the Guide for the Care and Use of Laboratory Animals organized by the National Academy of Sciences, and all efforts were made to decrease mice suffering and the number of experimental mice.

#### 2.3.2. Preparation of Alloxan-Induced Diabetic Mice

In this study, mice received an intraperitoneal cure with alloxan formulated in sterile normal saline with 1% (*m*/*v*) at 150 mg/kg body mass [37]. The control group got the similar volume of sterile normal saline. Animals were fasted for 14 h, but water was delivered without restrictions before treatment. Later, animals were kept in surveillance for 3 days. Serum glucose was revealed by the glucose oxidase peroxidase technique using a glucometer (One Touch Ultra, LifeScan, Milpitas, CA, USA). Mice with plasma glucose levels that exceeded 200 mg/dl were involved in the experiment.

**Figure 2:**
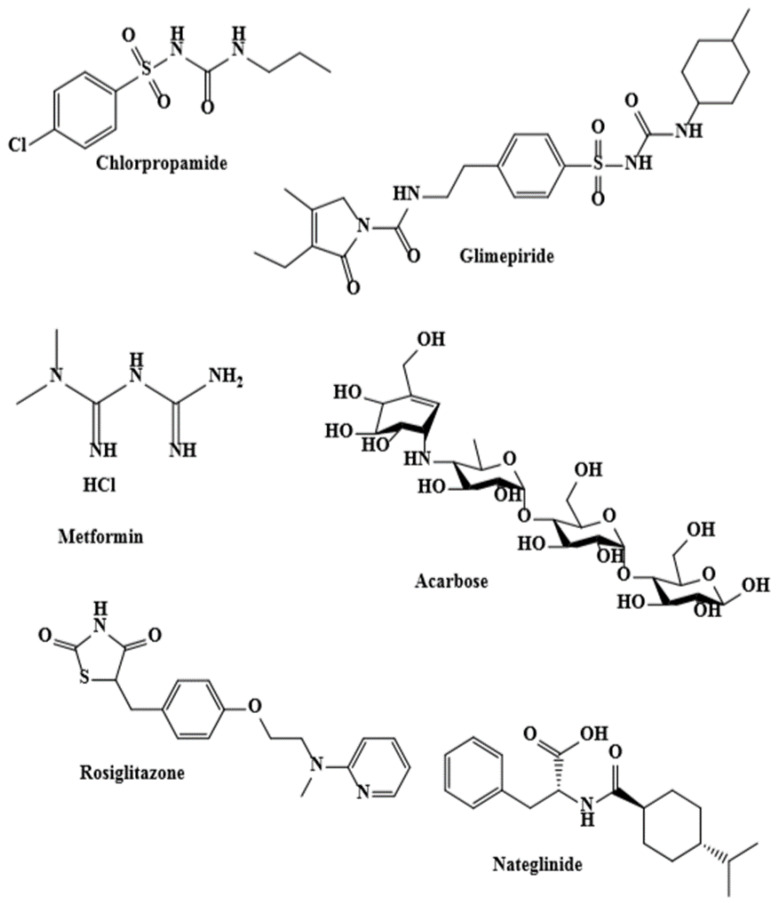
Representative oral synthetic antidiabetic agents [40]. Copyright: © 2014 Singab AN, et al.

In accordance with previously conducted studies [6,35,38,39,40,41], metformin was used as a hypoglycemic agent (Figure 2) because it is the best generally utilized glucose-decreasing treatment for T2DM. It considerably reduces blood glucose concentrations and improves unusual water drinking behavior in diabetic animals [38,42].

#### 2.3.3. Evaluation of Anti-Hyperglycemic Effect

In our study, the mice were divided into five groups of six mice each. The first group with normal mice obtained everyday distilled water. Group II with diabetic mice also obtained everyday distilled water. The third group with diabetic animals was cured with the experimented aqueous saponin extract with 100 mg/kg per os. Group IV with diabetic mice received Argan oil with 3 mL/kg per os. Group V with diabetic mice was treated by gastric gavage with metformin (300 mg/kg per os [39]. Several studies have adopted per os this dose of metformin [39,43]. All animals were cured for one month. The body weight and glycaemia were evaluated every 7 days. Food and water intake, and urinary volume were revealed for the first and last day using metabolic cages. After finishing the treatment, mice were killed for the evaluation of biochemical parameters.

#### 2.3.4. Blood Experiments

On day 30, blood samples were collected by using capillary tubes containing ethylenediaminetetraacetic acid (EDTA) (anti-coagulant). The blood tests were taken by the retro-orbital plexus puncture method. Centrifugation of blood samples occurred at 3000 rpm for 10 min at 4 °C, and the obtained plasma was collected for analysis.

#### 2.3.5. Biochemical Parameters

The following biochemical parameters were estimated in the collected blood samples: creatinine, serum glucose, aspartate amino transferase (ASAT), triglycerides, uric acid, total protein, urea, cholesterol, and alanine aminotransferase (ALAT). These levels were determined by an Abbott Architect c8000 model auto-analyzer (Abbott Park, Illinois, USA) according to the manufacturer’s instructions.

### 2.4. Statistic and Software

Data were stated as mean values ± standard deviation (SD) for each series of measurements. The significance of differences between multiple averages was determined by a one-way analysis of variance (ANOVA), and a Tukey’s post hoc test at a 5% level. The analysis was presented with Graphpad Prism 6.0.

## 3. Results

### 3.1. Nutritional Quality Assessment of Hand Pressed Argan Oil

The oil is rich in mono-unsaturated fatty acids (MUFA, 45%), which are mainly composed of oleic acid (C18:1, 31%) and gadoleic acid (C20:1, 0.19%). The poly-unsaturated fatty acids (PUFA, 31%) are from the second abundant fraction in the oil. They are the sum of linoleic acid (C18:2, 31%) and linolenic acid (C18:3, 0.19%). The saturated fatty acids (SFA, 20%) are the minor fraction and are composed of palmitic acid (C16:0, 13%), stearic acid (C18:0, 6%), arachidic acid (C20:0, 0.23%), and myristic acid (C14:0, 0.16%). Both MUFA/PUFA and PUFA/SFA ratios were above 1.5, which justify the oil freshness and nutritional quality. Those ratios are applied to assess the nutritional value and shelf-life stability of olive oils [44]. A PUFA/SFA ratio above 1.5 is related to nutritional and health benefits [45]. The sterol profile, measuring individual sterols, such as schottenol, spinasterol, Δ-7-avenasterol, stigma-8-22-dien-3β-ol, cholesterol, and campesterol, was also determined (Table 2). The total sterol content is very high with 209 mg/100 g of oil, with schottenol (46%), followed by spinasterol (39%), Δ-7-avenasterol (6%), stigma-8-22-dien-3β-ol (4%), cholesterol (0.32%), and campesterol (0.31%) forming the main sterols. On the other hand, the oil is also highly rich in tocopherols with 870 mg/kg oil of total tocopherols, composed of γ-tocopherol (721 mg/kg oil), δ-tocopherol (94 mg/kg oil), α-tocopherol (53 mg/kg oil), and β-tocopherol (3 mg/kg oil) (Table 2).

The total polyphenol content exhibited a concentration of (Gallic-Acid Equivalents) 113 mg GAE·kg^−1^ oil, which is a moderate concentration (Table 2).

The polyphenolics were quantified and identified by the HPLC-DAD-TOF/MS system. The reported results (Table 2) indicated the occurrence of ten phenolic compounds. Ferulic-acid showed the highest content (6.34 mg/kg), followed by syringic-acid (4.76 mg/kg), p-hydroxybenzoic acid (2.80 mg/kg), vanillic-acid (2.07 mg/kg), caffeic-acid (1.71 mg/kg), p-coumaric acid (0.87 mg/kg), gallic-acid (0.53 mg/kg), sinapic-acid (0.43 mg/kg), quercetin (0.38 mg/kg), and epicatechin (0.34 mg/kg).

### 3.2. In Vitro Anti-Diabetic Effect

#### Inhibitory Effects of Glucosidase and Amylase

**Figure 3:**
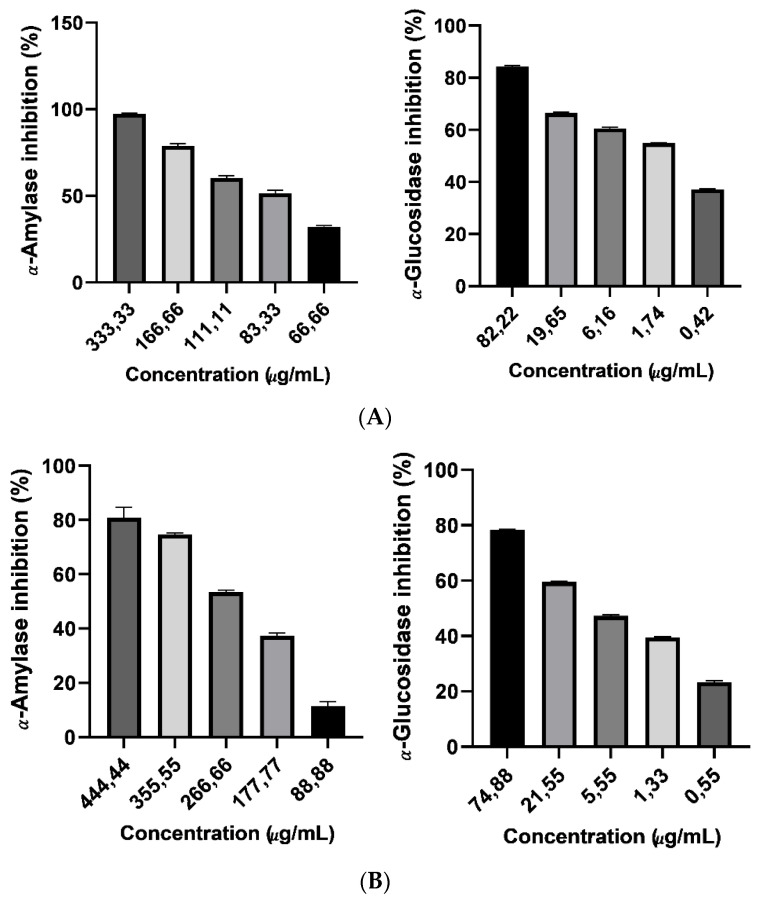
Average percentage of α-glucosidase and α-amylase inhibition versus concentration of Argan oil (**A**) and saponin Argan cake (**B**).

The α-amylase inhibitory activities of the Argan oil and saponin cake extract is introduced in Figure 3. The result showed that the experimented extracts inhibited α-amylase activity dosage dependently of (66.66–333.33 μg/mL) and (88.88–444.44 μg/mL), respectively. Moreover, all extracts indicated significantly (*p* < 0.05) more activity than the acarbose (IC50 = 310.10 ± 0.22 μg/mL) (Table 3). The Argan saponin cake extract has a better inhibitory effect versus α-amylase with IC50 value of 209.10 ± 0.17 μg/mL. Similarly, the extracts have proved encouraging and concentration-dependent (0.55–74.88 μg/mL) inhibitory activities on α-glucosidase enzyme (Figure 3A). Curiously, the IC50 values 0.89 ± 0.17 μg/mL, 7.56 ± 0.38 μg/mL for saponin extract and Argan oil, respectively, show that all examined extracts were significantly (*p* < 0.05) greater inhibitors of α-glucosidase than the acarbose (IC50= 17.02 ± 1.22 μg/mL) (Table 3).

### 3.3. In-Vivo Antidiabetic Activity

#### 3.3.1. Acute Oral Toxicity and Anti-Hyperglycemic Effect

Animals treated with saponin extract showed a DL50 of 1300 mg/kg for the oral route during the acute toxicity study [17].

The effects of the aqueous saponin Argan cake extract, Argan oil, and metformin, are shown in Table 4. After 7 days, there was no statistical distinction between the blood glucose concentration of the diabetic mice treated with the studied saponin extract, Argan oil, or metformin and diabetic control mice (untreated) (*p* > 0.05). After 14 days of therapy with aqueous saponin extract, a considerable reduction in blood glucose concentration was detected (*p* < 0.05); the showed glycemia value was comparable to that shown by the metformin-cured mice but still better than that of the normal mice. After 30 days, the blood glucose concentration of the mice cured with the saponin extract had reduced to reach that of the normal control mice and the metformin-cured mice. The aqueous saponin extract thus applied an antidiabetic activity identical to that of metformin and better than that of Argan oil [35].

Table 5 shows the effect of the daily administration of saponin Argan cake extract and Argan oil on urinary volume, food, and water intakes in the different mice groups.

Compared to the DC group, urinary volume, food, and water intake in the treated groups reduced significantly (*p* < 0.05).

#### 3.3.2. Consequences on Body Weight

The average body weight in all mice groups on days 7, 14, and 30 of therapy is presented in Table 6. After 7 and 14 days, no differences were detected between the groups (*p* > 0.05). On day 30, a significant reduction in the body mass of untreated diabetic mice (from 33.61 ± 2.14 g on day 7 to 24.35 ± 2.23 g on day 30) was detected related to the normal mice (Tukey’s test, *p* < 0.05). Compared to their initial weight, the diabetic control animals experienced significant weight loss (27.55%) over the experimental period, while normal animals increased weight (12.49%), and compared to their initial weight cured animals had constant weight. The group of diabetic mice cured with aqueous saponin extract and those treated with metformin showed a constant body weight between day 7 to day 30; both groups of cured mice had a relatively consistent weight. Moreover, as seen in Table 6, a comparison of the body weights in all groups on day 30 reveals significant differences between the untreated diabetic mice and the groups cured with the saponin cake extract and metformin (Tukey’s test, *p* < 0.05) [35]. The group of diabetic animals treated with Argan oil had an intermediate body weight.

Induction of diabetes with alloxan was thus associated with the characteristic development of hyperphagia (Tukey’s test, *p* < 0.05) (Table 5), polydipsia (Tukey’s test, *p* < 0.05) (Table 5), and decrease of body weight (Tukey’s test, *p* < 0.05) (Table 6).

#### 3.3.3. Consequences on Biochemical Parameters

Medicament utilized in the therapy of T2DM are anticipated to influence biochemical parameters; triglycerides, ALAT, uric acid, ASAT, and total cholesterol [35]. The activities of the saponin cake aqueous extract on these biochemical parameters were also examined (Table 7). Urea, total cholesterol, uric acid, and total protein showed statistical difference between the groups (*p* > 0.05). The highest creatinine levels (5.85 mg/L) were by the diabetic control group. Mice treated with the saponin cake extract and Argan oil presented inferior levels of creatinine. Consequently, weight loss may be associated to creatine dehydration in muscle cells that generate creatinine, as this will be eliminated in the urine.

Regarding the triglyceride concentration, untreated diabetic animals revealed the highest concentration (1.30 g/L). The triglyceride concentration in the mice cured with the saponin cake extract (0.71 g/L) is statistically comparable to that of the normal control (0.69 g/L) (Tukey’s test, *p* < 0.05).

Concerning the ASAT level, the findings for the normal control and the diabetic animal cured with the saponin extract were approximately similar (139 and 136 UI/L, respectively). Nevertheless, metformin-cured mice had a better concentration than these 2 groups. The ASAT concentration of the diabetic control animal was the greatest among the groups with a value of 379 UI/L [35].

Likewise, the ALAT concentrations of the normal control and the diabetic animal cured with the saponin extract exhibited similar concentrations (42.17 and 43.10 UI/L, respectively). The diabetic mice showed the greatest ALAT concentration [35].

This permitted us to show an anti-hyperglycemic action present in saponin-rich cake fractions, which supports the traditional use of Argan almonds against T2DM.

## 4. Discussion

The regular consumption of Argan oil could protect the human body from cancer and heart diseases [46]. The quality and purity of the hand pressed Argan oil were tested and investigated measuring 35 chemical parameters (Table 2).

The Argan oil has a specific chemical composition, which contributes to its uniqueness and its therapeutic effects. Its sensory profile and chemical composition lead to the classification of it in different Argan oil categories, including extra virgin, virgin, and lampante oil as described in the Moroccan Official Guidelines [47]. The oil category and freshness is certified based on parameters that include index of acidity (0.3%), peroxide value (PV, 2.3 meq O_2_/kg), and UV absorbances coefficients K232 (1.38) and K270 (0.27). From those parameter values, the applied oil is classified as extra virgin [47].

The fatty acids, sterols, and tocopherol contents of the oil are within the extra virgin category limits [47]. Previous studies conducted on Argan oil determined some factors that could influence its chemical composition, such as processing and extraction practices [48], roasting processes [49], ripeness and/or post-harvests of the Argan fruits [50] and geographical origin [24]. The extraction process of Argan oil (mechanical or traditional pressing) significantly influences the total polyphenol contents [7]. In addition, Argan oil shows the highest total polyphenol content in comparison to other vegetable oils [51]. Hand pressed Argan oil was reported to have a higher polyphenol content than mechanically pressed [7]. Moreover, the phenolic profile of the individual compounds varied significantly between both oil extractions (mechanical vs. hand processed) [40,52,53,54].

The hand pressed Argan oil showed a good and balanced chemical profile which needs more advanced investigations.

Numerous plant-resulting products are rich in bioactive polyphenols, of which some present a powerful anti-hyperglycemic property or health-promoting properties [7].

This study informs about the inhibitory kinetics of the Argan saponin cake extract and Argan oil anti-key enzymes (α-amylase and α-glucosidas) associated to hyperglycemia. Compared to the positive control, the in-vitro test showed a modest inhibitory activity. Considering the safe profile of Argan saponin cake extract, it can be of importance to consider an extract formulation for inhibition of (α-amylase and α-glucosidas) key enzymes in the small bowel. Such extracts may show an anti-hyperglycemic activity and be utilized as a capsulated formulation. The existence of various compounds in the extract, e.g., Arganin A, B, C, D, E, and F and Mi-Saponin A [20], results in a varied form of inhibition. In therapeutic strategies, many bioactive compounds together could be much more active than the individual compounds.

Inducing diabetes in an animal model by alloxan provides knowledge regarding the pathologic mechanism of diabetes, and is also used to screen treatments for diabetes and diabetes-associated problems. In this study, the potential hypoglycemic activities of Argan saponin cake extract and Argan oil treatments were studied using alloxan-induced diabetic mice.

In reality, the interaction between deficiencies in insulin emission and insulin effect give the heterogeneous character of type 2 diabetes. Many body systems are damaged because of the insufficiency resulting from improved concentrations of blood glucose [54]. Thus, for good diabetes management, there is a requirement to control postprandial blood glucose. The inhibition of key digestives is one of the approaches for glucose management [55], which gives a considerable decrease of the post-prandial blood glucose [56]. Some studies examined natural nutritional sources as choices with negligible side impacts and minimal therapy fees, despite the availability of glucosidase inhibitors [57]. Regardless of the efficacy of acarbose as an anti-hyperglycemic treatment with the inhibitory effect of glucosidase, food choices are required because of the side effect of acarbose [58]. Many studies about functional foods have been conducted [59]. A research paper described the amylase-inhibitory activities of pulp, leaf, and seed extracts of two Argan varieties [60].Yet, no previous study has revealed the kinetics and concentrations of this inhibition. The reported studies did not particularly examine the Argan cake saponin extract, they only focused on entire sets of phenolic compounds. The Argan anti-enzymatic activity is possibly partly associated to its cake saponin content. As we approved a nutritional therapy with (analgesic, antiradical, anti-inflammatory, and antidiabetic) bioactivities [23], the safe Argan cake saponin extracts were selected for in-vitro and in-vivo activity studies. The capacity to decrease the post-prandial rise of blood glucose levels was demonstrated by the inhibitory activity of the extract and Argan oil against the enzymes α-amylase and α-glucosidase. Our conclusion is in accord with previous articles that confirmed that some extracts of bioactive medicinal plants have more extra powerful α-glucosidase inhibitory activities than potent synthetic inhibitors [34,61]. Saponin compounds were reported to exhibit a glucosidase and amylase inhibitory effect [62,63,64,65]. The findings are also linked with a report concerning the insulin-sensitizing activities of *Argania spinosa* seed extracts that showed an insulin-sensitizing activity in the saponin-rich press cake fractions, and this gives support to the traditional utilization of Argan almonds against T2DM [66].

Recently, we have reported the in-vivo anti-inflammatory activity and the bioactive compounds’ profile of the polyphenolic extract from edible Argan oil acquired by two extraction techniques [7], who have completed previous studies, i.e., on the in-vivo anti-inflammatory activity of argan oil [27], the in-vivo acute and chronic toxicity of saponins from *Argania spinosa* [20], and the in-vivo anti-inflammatory and analgesic actions of *Argania spinosa* saponins [26].

Alloxan at 150 mg/kg damages the pancreatic β-cells. More quantities of glucose in the blood result of the insufficient secretion of insulin [42]. The hypoglycemic activities of Argan oil, 3 mL/kg per os, was shown to have the ability to increase the hepatic glycogen levels in diabetic hypertensive rats [67].

Moreover, Argan oil considerably decreased the quantity of absorbed glucose in a perfused jejunum segment relative to controls rats [68].

Another study about insulin-sensitizing seed extracts of *Argania spinosa* demonstrated that a saponin cake subfraction enhanced insulin-induced protein kinase B activation [66].

Previous work demonstrated an anti-inflammatory effect, like that of indomethacin that occurred within one month in animals treated by 100 mg /kg per os of Argan cake saponins [20], and it was reported that the hypoglycemic effect might be associated to improve transportation of the glucose into adipose, which is stimulated via the phosphatidyl-inositol-3-kinase pathway [66]. Manifestly, many papers described the hypoglycemic effects of the triterpenic type saponins. Matsuda and Yoshikawa [69] have described that with the presence of olean-12-ene 3 and 28-acylated bidesmoside have been the composition requirements for hypoglycemic action. There is a reduction of the transmission of glucose from the stomach to the small intestine by these saponins [70,71], a reduction of the glucose transfer at the brush border of the intestinal lining [72,73], and a decrease of serum glucose levels in glucose-loaded mice.

The reduction of the body weight in diabetic mics was attached to the loss in muscle mass [41]. The muscle weight and the kidneys’ rejection capability determined the blood creatinine concentration, but the rise of mice blood creatinine may be explained by the loss of mass detected in untreated animals [35,63]. Alternatively, for identifying toxic activities associated to therapy with compounds in mice, the concentration of creatinine is regularly judged to be a clinical factor [74]. The diabetic animals cured with Argan cake saponins and Argan oil were observed for the period of four weeks, and a considerable decrease in creatinine concentration was confirmed. Hence, two suggestions can be possible: Argan cake saponins and Argan oil reduced creatine catabolism and phosphocreatine catabolism in animal muscles or increased renal variations in diabetic animals.

Then again, the evolution in urinary quantity, food and water intake in diabetic mice is similar to the polyphagia and polydipsia detected in diabetic patients [75]. Argan therapy reduced diabetic signs. After a therapy phase, the Argan saponin cake capacity to inhibit key enzymes could be responsible for the reduction of glucose levels in serum. Argan cake saponin therapy decreased the total cholesterol and serum TG in an alloxan-induced diabetic animal. As a result, the regulation of blood lipid abnormalities and the decrease of probability of atherosclerosis can be produced by Argan intake therapy. Accordingly, for better liver function, the ALAT and ASAT are responsible markers [76]. The progress in ALAT and ASAT actions in plasma, in the alloxan model of diabetes, shows the liver necrosis and the alloxan hepatotoxic action [77]. Argan cake saponins and Argan oil therapies demonstrated their hepatoprotective impact, and decreased theses enzymes concentrations in plasma associated to the DC and therefore avoided the liver destruction.

Metformin decreases glucose creation via the suppression of gluconeogenesis in the liver. Furthermore, it enhances the insulin control by endogenous glucose production and reduces the intestinal glucose absorption to a less significant level.

In this study, the safety of pancreatic β-cells seemed more related with the bioactive compounds from the Argan saponin cake than from Argan oil. In alloxan-induced diabetic mice, Argan saponin cake therapy reduced plasma glucose concentration, polydipsia, hyperphagia, and body mass. Argan saponin cake extract and Argan oil intakes also showed an improvement of glucose tolerance. Furthermore, Argan cake intake avoided hypercholesterolemia and atherosclerosis in the diabetic animals.

In summary, the results encourage the theory that Argan cake saponins could successfully improve the symptoms of diabetes and regulate glucose metabolism. As a consequence, Argan cake intake is good for both patients with T2DM and healthy subjects.

This study confirmed the natural nutritional benefit of Argan oil and Argan cake saponins as a food and investigated the effect of Argan on selected digestive enzymes; the anti-enzymatic activity is possibly partly associated to its cake saponin content. We approved a nutritional therapy with anti-hyperglycemic bioactivity.

Focusing on their pharmacological and agri-food valorization, the present research sought and compared the inhibition of digestive enzymes of Argan cake saponins and traditional Argan oil. However, various individual elements of *Argania spinosa* seeds might be reliable to the consequences noted in this experiment, and antagonism or synergy between components is also probable. Thus, we can consider the possibility of introducing argan products in the food compliment formation.

## Figures and Tables

**Table 1:** Structure of the mice diet.

Component	Proportion (g/ 100 g Diet)
Corn flour	30
Wheat flour	21
Soybean cake	24
Wheat bran	5
Sorghum flour	8
Fishmeal	5
Bone powder	2
Yeast powder	3
Mineral mixture	0.7 (mg)
Salt	1
Vitamin mixture	0.3 (mg)
Total	100

**Table 2:** The chemical composition of hand pressed Argan oil showed as mean ± standard deviation (SD).

	EVAO	Argan Hand Pressed Oil		
Chemicals	Regulation	Mean ± SD (*n* = 3)	Min	Max
Quality Indices				
Acidity (%)	≤0.8	0.30 ± 0.01	0.28	0.32
Peroxide value (meq O_2_/kg)	≤15	2.3 ± 0.2	2.1	2.4
K_232_	≤2.52	1.38 ± 0.04	1.35	1.42
K_270_	≤0.35	0.27 ± 0.03	0.25	0.30
Fatty Acids (% of Total Fatty Acid)				
C_14:0_	≤0.20%	0.16 ± 0.02	0.14	0.17
C_16:0_	11.50–15.00%	13.43 ± 0.21	13.20	13.60
C_18:0_	4.30–7.20%	5.74 ± 0.06	5.69	5.80
C_20:0_	≤0.50%	0.23 ± 0.03	0.20	0.25
C_18:1_	43.10–49.00%	45.21 ± 0.31	44.90	45.52
C_20:1_	≤0.50%	0.23 ± 0.03	0.20	0.25
C_18:2_	29.30–36.00%	30.50 ± 0.30	30.20	30.80
C_18:3_	≤0.30%	0.19 ± 0.04	0.15	0.22
SFA	--	19.56 ± 0.30	19.23	19.82
MUFA	--	45.44 ± 0.34	45.10	45.77
PUFA	--	30.69 ± 0.34	30.35	31.02
MUFA/PUFA ratio	--	1.48 ± 0.01	1.48	1.49
PUFA/SFA ratio	--	1.57 ± 0.01	1.57	1.58
Tocopherols (mg/kg Oil)				
α-tocopherol	18–75	52.84 ± 0.49	52.50	53.40
β-tocopherol	1.0–5.0	2.60 ± 0.26	2.40	2.90
ɣ-tocopherol	640–810	721.00 ± 3.61	718.00	725.00
δ-tocopherol	54–110	93.56 ± 1.31	92.62	95.05
Total tocopherol	600–900	870.00 ± 5.44	865.52	876.35
Sterols (% of Total Sterols)				
Cholesterol	≤0.40	0.32 ± 0.02	0.30	0.33
Campesterol	≤0.40	0.31 ± 0.02	0.30	0.34
∆^7^-Avenasterol	4.00–7.00	5.73 ± 0.11	5.60	5.80
Stigma-8-22-dien-3β-ol	3.20–5.70	3.69 ± 0.17	3.50	3.80
Schottenol	44.00–49.00	46.42 ± 0.10	46.30	46.50
Spinasterol	34.00–44.00	38.80 ± 0.30	38.50	39.10
Total sterols (mg/100 g oil)	≤220	208.72 ± 1.26	207.80	210.15
Total Polyphenols (mg GAE·kg^−1^)		112.67 ± 2.55	110.20	115.30
Phenolic Compounds (mg·kg^−1^)				
Gallic-acid	--	0.53 ± 0.03	0.49	0.55
p-Hydroxybenzoic-acid	--	2.80 ± 0.05	2.75	2.85
Vanillic-acid	--	2.07 ± 0.10	1.95	2.15
Caffeic-acid	--	1.71 ± 0.04	1.68	1.75
Syringic-acid	--	4.76 ± 0.09	4.68	4.85
p-Coumaric-acid	--	0.87 ± 0.06	0.82	0.93
Ferulic-acid	--	6.34 ± 0.10	6.22	6.40
Sinapic-acid	--	0.43 ± 0.05	0.38	0.47
Epicatechin	--	0.34 ± 0.04	0.29	0.36
Quercetin	--	0.38 ± 0.03	0.35	0.40

EVAO, Extra Virgin Argan Oil; MUFA, Mono-Unsaturated Fatty Acids; PUFA, Poly-Unsaturated Fatty Acids; and SFA, Saturated Fatty Acids; GAE, gallic-acid equivalents; “--”, no regulation is defined.

**Table 3:** IC50 values of saponin Argan cake extracts and Argan oil on α-glucosidase inhibition and α-amylase.

	IC50 (µg/mL)	
	α-Amylase	α-Glucosidase
Acarbose	310.10 ± 0.22 ^b^	17.02 ± 1.22 ^c^
Saponin cake	209.10 ± 0.17 ^a^	0.89 ± 0.17 ^a^
Argan oil	597.10 ± 0.26 ^c^	7.56 ± 0.38 ^b^

The values are the mean of three determinations ± standard deviation. Values in the similar column with a different letter (a–c) vary significantly (Tukey’s test, *p* < 0.05).

**Table 4:** The effects of aqueous saponin Argan cake extract and Argan oil on fasting blood glucose levels in alloxan-induced diabetic mice.

	Fasting Blood Glucose Level (mg/dL) (*n* = 6)		
5 Groups	7th Day	14th Day	30th Day
Normal control	98.74 ± 4.40 ^a^	100.55 ± 4.33 ^a^	99.4 ± 0.95 ^a^
Diabetic control	240.22 ± 10.62 ^b^	274.33 ± 17.31 ^d^	200.70 ± 15.21 ^c^
Diabetic + Aqueous saponin cake extract (100 mg/kg per os)	238.14 ± 24.85 ^b^	136.41 ± 20.42 ^b^	106.55 ± 3.15 ^b^
Diabetic + Argan oil (3 mL/kg per os)	249.33 ± 11.64 ^b,c^	200.17 ± 39.61 ^c^	195.83 ± 7.15 ^c^
Diabetic + metformin (300 mg/kg per os)	253.17 ± 29.47 ^c^	143.83 ± 20.27 ^b^	104.67 ± 4.08 ^a,b^

^a–d^ Means within the same column: different letters are statistically significant (Tukey’s test, *p* < 0.05).

**Table 5:** Changes in metabolic parameters (food intake, water intake, and urinary volume in groups).

Parameter		NC	DC	D-Met	D-Saponins	D-Argan Oil
Food intake (g)	D1	5.24 ± 0.51 ^a^	9.38 ± 0.95 ^b^	7.30 ± 0.11 ^a^	7.98 ± 0.19 ^b^	8.5 ± 0.65 ^b^
	D30	6.77 ± 0.11 ^a^	13.25 ± 0.65 ^c^	8.90 ± 0.13 ^a^	9.80 ± 0.45 ^b^	10.76 ± 1.03 ^b^
Water intake (mL)	D1	4.29 ± 0.32 ^a^	11.13 ± 1.02 ^c^	8.24 ± 0.81 ^b^	9.04 ± 0.77 ^b^	10.01 ± 0.98 ^b^
	D30	5.1 ± 0.41 ^a^	27.48 ± 1.45 ^d^	10.78 ± 1.2 ^b^	13.1 ± 0.91 ^b^	18.55 ± 1.5 ^c^
Urinary volume (mL)	D1	1.5 ± 0.22 ^a^	6.7 ± 0.4 ^b,c^	5.3 ± 0.33 ^b^	4.7 ± 0.77 ^b^	5.5 ± 0.41 ^b^
	D30	1.8 ± 0.35 ^a^	10.1 ± 1.3 ^d^	6.4 ± 0.45 ^b^	6.7 ± 0.43 ^b^	7.1 ± 0.85 ^b,c^

Data reported are mean ± SD (*n* = 6). Values in the same column not sharing a common letter (a–d) differ significantly (Tukey’s test, *p* < 0.05).

**Table 6:** The average body weight of mice in the different groups.

	Body Weight, ±SD (g) (*n* = 6)		
Groups	7th Day	14th Day	30th Day
Normal control	32.36 ± 1.55 ^a^	34.11 ± 0.44 ^b^	36.98 ± 0.75 ^d^
Diabetic control	33.61 ± 1.64 ^a^	30.70 ± 1.68 ^a^	24.35 ± 1.10 ^a^
Diabetic + Aqueous saponin extract (100 mg/kg per os)	33.71 ± 1.15 ^a^	33.31 ± 1.59 ^b^	33.45 ± 1.21 ^c^
Diabetic + Argan oil (3 mL/kg per os)	33.18 ± 1.12 ^a^	31.80 ± 1.46 ^a^	29.99 ± 1.25 ^b^
Diabetic + Metformin (300 mg/kg per os)	32.88 ± 1.17 ^a^	31.95 ± 1.23 ^a^	32.55 ± 1.44 ^c^

^a–d^ Different letters within the same column are statistically significant (Tukey’s test, *p* < 0.05), ±standard deviation (SD).

**Table 7:** Effects of saponin cake aqueous extract and Argan oil on biochemical parameters in alloxan-induced diabetic mice.

Parameter	Normal Control	Diabetic Control	Diabetic + Aqueous Saponins Cake Extract (100 mg/kg Per os)	Diabetic + Argan Oil (3 mL/kg Per os)	Diabetic + Metformin, (300 mg/kg Per os)
Total protein, g/L	63.22 ± 1.33 ^b^	58.26 ± 1.44 ^a,b^	63.67 ± 2.51 ^b^	60.95 ± 1.45 ^b^	56.09 ± 1.58 ^a^
Urea, mg/dL	0.30 ± 0.01 ^b^	0.34 ± 0.01 ^c^	0.25 ± 0.01 ^a^	0.26 ± 0.01 ^a^	0.30 ± 0.02 ^b^
Cholesterol, g/L	0.46 ± 0.05 ^a^	1.10 ± 0.05 ^c^	0.49 ± 0.08 ^b^	0.51 ± 0.02 ^b^	0.47 ± 0.03 ^a,b^
Creatinine, mg/L	3.72 ± 0.05 ^a,b^	5.85 ± 0.14 ^c^	3.72 ± 0.02 ^a,b^	3.76 ± 0.04 ^b^	3.55 ± 0.03 ^a^
Triglyceride, g/L	0.69 ± 0.08 ^a^	1.30 ± 0.05 ^c^	0.71 ± 0.04 ^a^	0.95 ± 0.05 ^b^	0.70 ± 0.04 ^a^
Uric acid, mg/L	26.01 ± 1.10 ^a,b^	25.30 ± 1.15 ^a^	26.33 ± 0.86 ^a,b^	26.75 ± 1.28 ^b^	29.99 ± 1.01 ^c^
ASAT, UI/L	138.55 ± 5.22 ^a^	378.88 ± 15.75 ^c^	136.36 ± 5.92 ^a^	136.30 ± 4.52 ^a^	280.321 ± 11.72 ^b^
ALAT, UI/L	42.17 ± 1.02 ^a^	300.27 ± 14.03 ^c^	43.10 ± 1.13 ^a^	43.70 ± 1.33 ^a^	49.59 ± 1.15 ^b^

^a–d^ Results within the same row with different letters are statistically significant (Tukey’s test, *p* < 0.05).

## Data Availability

Data is contained within the article and available by emailing.
